# The synaptic correlates of serial position effects in sequential working memory

**DOI:** 10.3389/fncom.2024.1430244

**Published:** 2024-07-15

**Authors:** Jiaqi Zhou, Liping Gong, Xiaodong Huang, Chunlai Mu, Yuanyuan Mi

**Affiliations:** ^1^School of Medicine, Chongqing University, Chongqing, China; ^2^College of Mathematics and Statistics, Chongqing University, Chongqing, China; ^3^Department of Psychological and Cognitive Sciences, Tsinghua University, Beijing, China; ^4^Department of Physics, South China University of Technology, Guangzhou, China

**Keywords:** sequential working memory, serial position effect, short-term plasticity effect, continuous attractor neural networks, the primacy and recency effect

## Abstract

Sequential working memory (SWM), referring to the temporary storage and manipulation of information in order, plays a fundamental role in brain cognitive functions. The serial position effect refers to the phenomena that recall accuracy of an item is associated to the order of the item being presented. The neural mechanism underpinning the serial position effect remains unclear. The synaptic mechanism of working memory proposes that information is stored as hidden states in the form of facilitated neuronal synapse connections. Here, we build a continuous attractor neural network with synaptic short-term plasticity (STP) to explore the neural mechanism of the serial position effect. Using a delay recall task, our model reproduces the the experimental finding that as the maintenance period extends, the serial position effect transitions from the primacy to the recency effect. Using both numerical simulation and theoretical analysis, we show that the transition moment is determined by the parameters of STP and the interval between presented stimulus items. Our results highlight the pivotal role of STP in processing the order information in SWM.

## 1 Introduction

Sequential working memory (SWM), a function responsible for temporarily storing and manipulating information in a specific order (Stephan and MJB, 1989; Jensen and Lisman, 2005; Logan, 2021), plays a fundamental role in brain cognitive functions, such as reasoning, comprehension and learning (Alan, 2003; Curtis and Lee, 2010; Potagas et al., 2011; Calmels et al., 2012; Ru et al., 2022). SWM supports human mental processes by providing an interface between perception, long-term memory and actions (Tsetsos et al., 2012). The memory recall paradigm is widely utilized to investigate the storage of multiple items in SWM (Endel and CFI, 2000; Pantelis et al., 2008; Henson, 2013), where subjects are required to retrieve previously presented information. A large volume of psychological experiments has demonstrated that the retrieval performances of human subjects are associated with the order of presented items, displaying a serial position effect, namely, subjects exhibit better performances for memory items appearing at the beginning or at end of a sequence, called the primacy or recency effect, respectively (Simon, 1962; Postman and Phillips, 1965; Glanzer and Cunitz, 1966). This serial position effect is observed in various types of working memory systems, including visual (Kiani et al., 2008), auditory (Hurlstone et al., 2014; Borderie et al., 2024), and spatial working memories (Groeger et al., 2008). The serial position effect is a well-established phenomenon in memory research, yet its underlying neural mechanism, contextual variation, and functional implication remain largely unresolved.

A number of psychophysical experiments have indicated that the serial position effect of SWM is affected by factors related to attention, context and interference. For instance, the attentional gradient, i.e., a gradual decrease in attention level as different items are presented during the encoding period, affects the primacy effect. Contents can also affect participants' retrieval performances, with the recency effect observed when the recall cue is the item order, while the primacy effect observed when the recall cue is the relative size of a specific feature of the item (Cowan et al., 2002; Li et al., 2021). Interference between items affects the recency effect (Gorgoraptis et al., 2011). Additionally, some variations in the experimental paradigm can affect the serial position effect, such as, increasing the inter-stimulus interval during the encoding can weaken the primacy effect but not the recency effect (Glanzer and Cunitz, 1966); prolonging the maintenance period in a visual sequence working memory task can shift participants' performances from the recency to the primacy effect (Knoedler et al., 1999), and this recency-primacy shift were observed in experiments including auditory, verbal, and text materials (Knoedler et al., 1999; Storm and Bjork, 2016). Finally, distractions at different time points during the maintenance period can lead to fluctuations in participants' retrieval accuracy (Lui et al., 2023).

Up to now, the neural mechanism underlying the serial position effect in SWM remains largely unclear. The perspective of “limited resource” proposed that the differential allocation of memory resources across multiple items governs their relative recall precision, thereby leading to the primacy and recency effects as observed in SWM tasks (Gorgoraptis et al., 2011; Ma et al., 2014; Lee et al., 2020; Wang et al., 2021). Another study proposed an attractor network model with firing rate adaptation, which explains the power law of recall capacity (Naim et al., 2020), as well as the primacy and recency effects in human free recall (Boboeva et al., 2021). Nevertheless, these studies did not explain how the detailed dynamics of a neural circuit account for the serial position effect. Recent studies have suggested that working memory is mediated by rapid transitions in “activity-silent” neural states (Wolff et al., 2017; Barbosa et al., 2020), and the strength of hidden-state representation predicts the accuracy of working memory-guided behavior, including recall precision, i.e., the primacy and recency effects (Stokes, 2015; Wolff et al., 2015, 2017; Katkov et al., 2017; Naim et al., 2020). The synaptic mechanism of working memory posits that information is encoded in the facilitated synaptic connections between neurons, rather than in the persistent responses of neurons (Mongillo et al., 2008; Mi et al., 2017). These works mainly studied the neural mechanism for storing and manipulating a single memory item. However, the neural circuit dynamics underlying the serial position effect during the storage of multiple (>2) memory items has not been investigated, which is the focus of the present study.

In this work, we adopt the view that information resides in hidden states of a neural circuit (Stokes, 2015) and is expressed by facilitated synapses between neurons (Mongillo et al., 2008). Specifically, we develop a model of continuous attractor neural network (CANN) with short-term synaptic plasticity (STP). Utilizing a delay task paradigm, we investigate the serial position effect in SWM. In the delay task, the whole period is divided into stimulus encoding, maintenance, and retrieval/response phases. During the encoding phase, participants sequentially receive and encode multiple items into their working memory. After a maintenance period, they are prompted to recall the items, with each item's recall performance indicating the precision of the corresponding memory representation. Typically, participants are required to retain a specific attribute of each item in the sequence, such as visual orientation, direction, or spatial location. Our model shows that with the prolongation of the maintenance period, the serial position effect gradually shifts from a significant primacy effect to a significant recency effect, with the recency effect diminishing in significance over time. This agrees well with the experimental finding. We further analyze that the transition moment of the serial position effect is predominantly determined by the STP dynamics and the inter-item interval of presenting stimuli. Our study highlights the important role of STP plays in processing the order information in SWM.

## 2 The model

**Figure 1:**
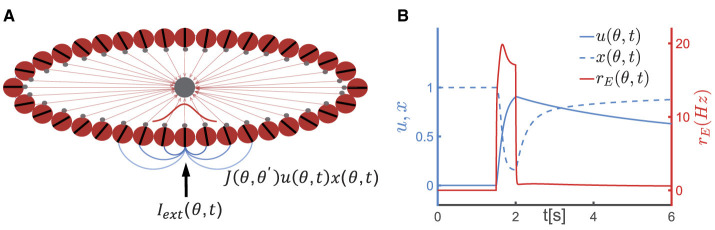
A continuous attractor neural network (CANN) model with short-term plasticity (STP). **(A)** The schematic diagram of the CANN. Excitatory neurons are arranged in a ring based on their preferred visual orientations θ (θ∈[−π^2^, π^2^)). The connection strength between two excitatory neurons at θ and θ′ is denoted as *J*(θ, θ′), which depends only on |θ−θ′| (the varying shades of gray lines represent the connection strength) and is translation-invariant in the feature space. All excitatory neurons in the network are connected to a global inhibitory neuronal pool (the gray node). The network generates a Gaussian-shaped bump (red curve) to represent the external stimulus (*I*_*ext*_(θ, *t*)) using population coding strategy. **(B)** The schematic diagram of STP. *u*(θ, *t*), *x*(θ, *t*) represent the release probability and the fraction of available neurotransmitters of neurons θ at time *t*, respectively. The firing rate of neurons at θ(*r*_*E*_(θ, *t*)) instantaneously increases (the red solid line) upon receiving external signals, leading to an increase in release probability of neurotransmitters (*u*(θ, *t*)) and a decrease in the fraction of available resources (*x*(θ, *t*)). Following the removal of the external stimulus, *u*(θ, *t*) decays to 0 within the time of τ_*f*_, and *x*(θ, *t*) returns to 1 within τ_*d*_. Due to the dominance of the synaptic short-term facilitation effect, *u*(θ, *t*) remains at a high level for an extended duration.

To elucidate the neural mechanism underlying the temporal dynamics of the serial position effect in SWM, we adopted a continuous attractor neural network (CANN) with short-term synaptic plasticity effect (STP). CANNs are a canonical model for neural information storage and representation (Wu et al., 2013, 2016) (Figure 1A), which has been successfully applied to describe the encoding of continuous features in neural systems, such as orientation (Ben-Yishai et al., 1995), moving direction (Georgopoulos et al., 1986), head direction (Taube et al., 1990), and spatial location of objects (Bottomley, 1987). Additionally, CANNs has been extensively used to model the neural mechanism of working memory (Mi et al., 2017; Li et al., 2021). STP is a ubiquitous phenomenon in neural systems, referring to the property that synaptic efficacy between neurons dynamically changes over time in a way that reflects the firing history of the pre-synaptic neuron (Figure 1B). Based on the property of STP, Mongillo et al. proposed a synaptic mechanism of working memory, stating that a neural circuit need not to maintain energy-intensive firings during the entire period of the task for memorizing stimuli, rather the neural circuit can utilize facilitated synaptic connections to retain information (Mongillo et al., 2008). The alteration in synaptic strength induced by STP is a relatively slow process that temporarily modifies the network's connectivity pattern, leading to the network's computation relying on the history of external inputs. Combining CANNs with STP, computational models have elucidated the maintenance and manipulation of working memory (Mi et al., 2017), and the phase precession phenomenon in hippocampus (Chu et al., 2022).

### 2.1 Continuous attractor neural networks

Consider a one-dimensional continuous stimulus θ, such as the visual orientation, is encoded by an ensemble of neurons. All excitatory neurons in the CANN (the red circles in Figure 1A) are aligned in a ring according to their preference under the periodic boundary condition, i.e., θ∈[−π/2, +π/2), and they are all reciprocally connected to a global inhibitory neuronal pool (the black circle in Figure 1A). Denotes *h*_*E*_(θ, *t*) as the synaptic inputs at time *t* of excitatory neurons at θ. The dynamics of *h*_*E*_(θ, *t*) is determined by a decay term, the recurrent current from other neurons, the inhibitory input from the global inhibitory neuronal pool and the external input, which is written as,


(1) $$\begin{array}{l} \;\tau \frac{\partial h_{E}(\theta ,t)}{\partial t}=-h_{E}(\theta ,t)\\ +\rho \int_{-\pi /2}^{\pi /2}J(\theta ,\theta^{\prime })u(\theta^{\prime },t)x(\theta^{\prime },t)r_{E}(\theta^{\prime },t)d\theta^{\prime }\\ \;-J_{EI}r_{I}+I_{ext}(\theta ,t)+I_{0}+\sigma_{0}\eta_{0}(\theta ,t), \end{array}$$

where τ denotes the time constant of neurons, ρ the neuronal density, *I*_0_+σ_0_η_0_(θ, *t*) the background input, with η_0_(θ, *t*) the Gaussian white noise of zero mean and unit variance and σ_0_ the corresponding noise strength. *I*_*ext*_(θ, *t*) refers to the external input, such as the visual stimulus during the encoding period and the cue during the recalling period. *J*_*EI*_ is the synaptic strength from the global inhibitory neuronal pool to excitatory neurons. *r*_*E*_(θ, *t*) is the firing rate of neurons with preference at θ, and its relationship with the synaptic current is given by *r*_*E*_(θ, *t*) = αln [1+exp(*h*_*E*_(θ, *t*)/α)], which is a smoothed threshold-linear function.

*J*(θ, θ′) is the synaptic connection between neurons at θ and θ′, as set in Equation 2,


(2) $$J(\theta ,\theta^{\prime })=\left\{ \begin{array}{l} J\cos[B(\theta -\theta^{\prime })],\;B(\theta -\theta^{\prime })\in \left[-\arccos(-J_{0}/J),\arccos(-J_{0}/J)\right]\\ \;J_{0}\;,\;else \end{array}\right.,$$

where *J*(θ, θ′) is a function of the difference in neuronal preferences (i.e., (θ−θ′)), which is translation-invariant in the feature space. Due to this characteristic topological structure, a CANN can hold a continuous family of stationary states, metaphorically understood as a valley of local minima in the network's energy landscape. *J* and *J*_0_ determine the synaptic connection strength between neurons, while *B* controls the synaptic interaction range. Neurons with similar preferences have stronger synaptic connections, while those with significantly different preferences have weaker connections.

The synaptic input to the global inhibitory neuronal pool is denoted as *h*_*I*_, with *r*_*I*_ the corresponding firing rate, and *r*_*I*_(*t*) = αln [1+exp(*h*_*I*_(*t*)/α)]. The dynamics of the global inhibitory neuronal pool is written as,


(3) $$\tau \frac{\partial h_{I}(t)}{\partial t}=-h_{I}(t)+J_{IE}\int_{-\pi /2}^{\pi /2}r_{E}(\theta^{\prime },t)d\theta^{\prime },$$

where τ denotes the time constant of the inhibitory neuronal pool, *J*_*IE*_ the connection strength from excitatory neurons in the ring to the inhibitory neuronal pool. The global inhibitory neural pool plays a crucial role in maintaining a balanced state between excitation and inhibition in the network, thereby preventing excessive neuronal firing. Additionally, it fosters competition among different groups of excitatory neurons, ensuring that only one memory item is represented at a given moment.

### 2.2 Short-term synaptic plasticity

Two types of STP, known as short-term facilitation (STF) and short-term depression (STD), have been observed in various cortical areas. STF is caused by the influx of calcium into the synaptic terminal of the pre-synaptic neuron following spike generation, which increases the release probability of neurotransmitters. On the other hand, STD is caused by the depletion of neurotransmitters at the synaptic terminal of the pre-synaptic neuron after spike generation.

In the model proposed by Mongillo et al. (2008), the STF effect is modeled by *u*(θ, *t*) (*u*∈[0, 1]), which indicates the release probability of neurotransmitters from pre-synaptic neurons at θ, and STD is modeled by *x*(θ, *t*)(*x*∈[0, 1]), indicating the fraction of available neurotransmitters in pre-synaptic neurons. The dynamics of STF and STD are given in Equation 4,


(4) $$\begin{array}{l} \begin{array}{cc} \frac{\partial u\left(\theta ,t\right)}{\partial t} & =-\frac{u\left(\theta ,t\right)}{\tau_{f}}+U_{0}\left(1-u\left(\theta ,t\right)\right)r_{E}\left(\theta ,t\right),\\ \frac{\partial x\left(\theta ,t\right)}{\partial t} & =-\frac{1-x\left(\theta ,t\right)}{\tau_{d}}-u\left(\theta ,t\right)x\left(\theta ,t\right)r_{E}\left(\theta ,t\right), \end{array} \end{array}$$

where τ_*f*_ and τ_*d*_ denote the time constants of STF and STD, respectively, and *U*_0_ the increment of *u* caused by spiking of the pre-synaptic neuron. When a neuron at θ receives external inputs, its firing rate (*r*_*E*_(θ, *t*)) increases. The increase in firing rate results in an increase in the release probability of neurotransmitter *u*(θ, *t*) (with the increment determined by *U*_0_), leading to the STF effect, while the proportion of available neurotransmitter *x*(θ, *t*) decreases, leading to the STD effect. Subsequently, *u*(θ, *t*) decays to its baseline of 0 with a time constant τ_*f*_, and *x*(θ, *t*) returns to its baseline of 1 with a time constant τ_*d*_, as illustrated in Figure 1B. The product of *u*(θ, *t*) and *x*(θ, *t*) represents the instantaneous synaptic efficacy at time *t*, i.e., *Ju*(θ, *t*)*x*(θ, *t*), which reflects the strength of memory representation in the network (Mi et al., 2017; Li et al., 2021).

To elucidate the neural mechanism of SWM, we selected parameters consistent with the synaptic connectivity between neurons in the prefrontal cortex (PFC), which is the primary crotical region involved in working memory (Wang et al., 2006). We adopted the STF dominant parameters as in the model proposed by Mongillo et al. (2008), i.e., τ_*d*_≪τ_*f*_ and a smaller *U*_0_. This implies that after neuron firing, the synaptic connection efficacy is maintained at a high value for an extended period to sustain memory information.

In our numerical simulation, we model the CANN by considering *N* neurons uniformly distributed in the range of [−π/2, π/2) in the feature space. The integration in Equation (1) is computed by,


$$\begin{array}{l} \int_{-\pi /2}^{\pi /2}J(\theta ,\theta^{\prime })u(\theta^{\prime },t)x(\theta^{\prime },t)r_{E}(\theta^{\prime },t)d\theta^{\prime }\\ =\frac{\pi }{N}\sum_{k=1}^{N}J(\theta ,\theta_{k})u(\theta_{k},t)x(\theta_{k},t)r_{E}(\theta_{k},t), \end{array}$$

The parameters are given in Supplementary Tables 1, 2.

## 3 Results

### 3.1 The serial position effect in SWM

**Figure 2:**
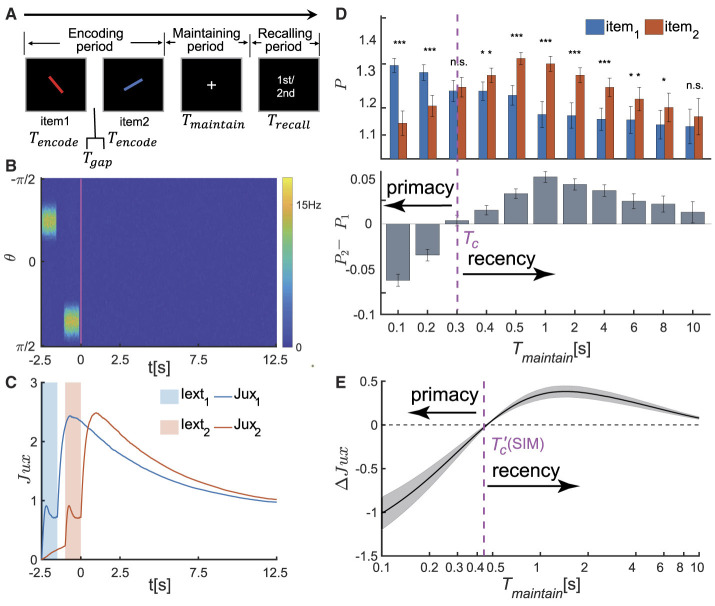
The serial position effect in SWM task for two items. **(A)** Schematic diagram of the psychophysical experiment paradigm. In the encoding phase, two distinct visual stimuli are sequentially presented. Each stimulus is presented for a duration denoted by *T*_*encode*_, separated by an time interval of *T*_*gap*_ between them. Following a delay period of *T*_*maintain*_, a recall cue lasting for *T*_*recall*_ is presented. Participates are then tasked with recalling the orientation value of either the first or second visual stimulus based on the given cue. **(B, C)** The temporal dynamics of neural activity pattern in an individual trial simulation example. **(B)** Two visual stimuli, denoted as θ_*i*_ (i=1,2), are presented sequentially during the encoding phase. In response to each stimulus, the network generates successive bump-shaped neural activity patterns centered at θ_*i*_, respectively. During the delay period, the neural activity gradually decays to a silent state. **(C)** The temporal course of synaptic efficacies of neural groups encoding two visual stimuli throughout the trail. When a stimulus is presented, the synaptic efficacy of the corresponding neural group rapidly decreases. After the stimulus is removed, the synaptic efficacy gradually recovers to a maximum value, denoted as $Jux_{i}^{max}$, within a certain period of time. Subsequently, it remains at a high level for an extended duration. **(D)** The recalling performance at varying *T*_*maintain*_. *T*_*c*_ denotes the critical moment of recall performance shift from primacy to recency effect, and *T*_*maintain*_∈{0.1*s*, 0.2*s*, 0.3*s*, 0.4*s*, 0.5*s*, 1*s*, 2*s*, 4*s*, 6*s*, 8*s*, 10*s*}. (Top) The normalized target probability of the *i*th presented item, denoted as *P*_*i*_ for *i* = 1, 2, at varying *T*_*maintain*_. (Bottom) The normalized target probability difference between the 1st and 2nd item (*P*_2_−*P*1) at varying *T*_*maintain*_. **(E)** The relative synaptic efficacy (Δ*Jux*(*t*)±SEM) of the neuronal groups encoding the two visual stimuli during maintaining period. $T_{c}^{\prime }\left(SIM\right)$ denotes the critical moment at which Δ*Jux*(*t*)≡0. The parameters settings see Supplementary Tables 1, 2. (n.s.: p > 0.05, *: 0.01 <p <0.05, **: 0.001 <p <0.01, ***: p <0.001).

Based on the above model of CANN with STP, we investigated the serial position effect in SWM. We first studied the case of two memory items and later generalized the study to the case of multiple items. We utilized the same paradigm as in the psychophysical experiments for SWM (Li et al., 2021), and investigated the recall accuracy of items based on their visual orientations, as illustrated in Figure 2A.

In each trail, two stimuli with different orientations (referred to as θ_1_, θ_2_) are sequentially presented in the encoding period. Following a delay period, a visual recall cue lasting for *T*_*recall*_ is presented, and the network retrieves either the first or second visual item based on the recalling cue. Let *T*_*encode*_ denote the duration of presenting each item, *T*_*gap*_ the time interval between two items, and *T*_*maintain*_ the duration of the delay period. The orientation values of two stimuli are set as: θ_1_ is randomly selected from the range [−π^2^, π^2^), and θ_2_ = θ_1_+Δθ, where Δθ is the difference between two stimuli, randomly selected from the data set [±17°, ±24°, ±38°, ±52°, ±66°, ±80°] (Li et al., 2021). The visual stimulus in the encoding period and the cues in the recalling period are denoted as *I*_*ext*_(θ, *t*), which are written as,


(5) $$\begin{array}{l} \;I_{ext}(\theta ,t)\\ =\left\{ \begin{array}{l} a_{type}(t)\cos[B_{type}\times (\theta -\theta_{type})]\\ \;,\;B_{type}\times (\theta -\theta_{i})\in [-\arccos(0),\arccos(0)]\\ \;+\sigma_{type}\zeta_{type}(\theta ,t)\;,\;\\ \;0,\;else \end{array}\right. \end{array}$$

where θ_*type*_(*type* = *encode, recall*) represents the visual stimulus during different periods. The parameters *a*_*type*_(*t*) and *B*_*type*_ regulate the strength and accuracy of external signals, respectively. A larger *a*_*type*_(*t*) and *B*_*type*_ result in more precise encoding of orientation information from the stimulus. As the recalling signals are unrelated to the task, the parameters are set as follows: *a*_*encode*_≫*a*_*recall*_, *B*_*encode*_>*B*_*recall*_ (Li et al., 2021).

When two visual stimuli are presented sequentially, the neural network generates successive bump-shaped neural activity patterns. The peaks of these bumps correspond to θ_1_ and θ_2_, respectively, as illustrated in Figure 2B. Due to the strong interactions among neurons with similar preferences and weak interactions among those with significantly different preferences in the network, we define a neuronal group *G*_*i*_ as the ensemble of neurons whose preferred values satisfy |θ−θ_*i*_| ≤ Δ (*i* = 1, 2), and this group of neurons primarily encodes the *i*th item. The corresponding neural activity and synaptic strength are calculated to be $r_{i}\left(t\right)=\frac{1}{m_{i}}\overset{\theta_{i}+\Delta }{\underset{\theta =\theta_{i}-\Delta }{\sum }}r_{E}\left(\theta ,t\right)$ and $Jux_{i}\left(t\right)=\frac{1}{m_{i}}\overset{\theta_{i}+\Delta }{\underset{\theta =\theta_{i}-\Delta }{\sum }}Ju\left(\theta ,t\right)x\left(\theta ,t\right)$, respectively, with *m*_*i*_ representing the number of neurons in *G*_*i*_. After removing stimuli, the firing rates of both neuronal groups gradually decrease to zero (Stokes, 2015; Wolff et al., 2015). However, their synaptic strengths remain at high levels due to STF, which maintain the stimulus information (see Figure 2C). During the recalling period, the network generates a weak bump-shaped activity pattern in response to the recalling cue, and the retrieved orientation (denoted as $\theta_{i}^{recalled}$, *i* = 1, 2) is decoded using the population vector method, with details given in the Supplementary material 1.1.

We investigated how the maintenance duration *T*_*maintain*_ affects the recall performance. We set *T*_*maintain*_ as a variable ranging from 0 to 10 s and selected 11 values within this range. For each chosen value of *T*_*maintain*_, we evolved the network for 50 times (corresponding to 50 different participants in a psychophysical experiment), each consisting of 300 trials. We utilized the normalized target probability method (Bays et al., 2009; Schneegans and Bays, 2016) to calculate the recall performance of each item (i.e.,$\theta_{i}^{recalled}$, *i* = 1, 2). More details see Supplementary material 1.2. We found that (as shown in Figure 2D):

- When *T*_*maintain*_ is smaller than a critical value denoted as *T*_*c*_, i.e., *T*_*maintain*_<*T*_*c*_, the recall performance exhibits the primacy effect, indicating that participates memorize the first item more accurately. Moreover, the primacy effect becomes more pronounced as the value of *T*_maintain_ decreases.
- When *T*_*maintain*_>*T*_*c*_, the recall performance shifts to the recency effect, indicating that participates memorize the second item more accurately. The significance of the recency effect gradually decreases as *T*_*maintain*_ increases.

We further utilized the methods of Circular Variance (CV) and Circular Kurtosis (CK; Berens, 2009) to calculate the accuracy of recall performance. The statistical results are consistent with those shown in Figure 2D (more details see Supplementary material 1.2 and Supplementary Figure 1). In conclusion, with the increase of the maintenance period, the serial position effect in SWM dynamically shifts from the primacy effect to the recency effect.

To further reveal the neural mechanism underlying the dynamical change of the serial position effects, we calculated the relative synaptic efficacy of two neuronal groups encoding two stimuli (θ_1_ and θ_2_) over time, denoted as Δ*Jux*(*t*) = *Jux*_2_(*t*)−*Jux*_1_(*t*) hereafter. The synaptic mechanism of WM posits that information is maintained in the facilitated synaptic interactions between neurons, with the synaptic efficacy determining the accuracy of the memorized item (Teyler and Discenna, 1984; Henry and Misha, 1996; Mongillo et al., 2008). For example, when two items (θ_1_ and θ_2_) are presented sequentially in a trail (Figure 2C), the transient synchronous firing of a neuronal group (*G*_1_ or *G*_2_, respectively) leads to rapid decrease in synaptic efficacy, due to the depletion of available neurotransmitters in neurons. After the visual stimulus disappears, the synaptic efficacy of the neural group recovers to the maximum value $\left(Jux_{i}^{max}\right)$ and maintains at a high level for an extended period. We calculated the synaptic efficacy between two neuron groups Δ*Jux*(*t*) during the maintaining period (Figure 2E) and found that:

- Since the second item is presented later, its synaptic efficacy *Jux*_2_(*t*) is smaller than that of the first one *Jux*_1_(*t*) before it recovers to the maximum value. Therefore, Δ*Jux*(*t*) <0 when $t<T_{c}^{\prime }\left(SIM\right)$, where $T_{c}^{\prime }\left(SIM\right)$ is the moment when Δ*Jux*(*t*)≡0.
- As the time *t* approaches $T_{c}^{\prime }\left(SIM\right)$, both Δ*Jux*(*t*) and its variance approach zero. When $t>T_{c}^{\prime }\left(SIM\right)$, Δ*Jux*(*t*) first increases and then gradually diminishes over time.

The critical moment $T_{c}^{\prime }\left(SIM\right)$ at which Δ*Jux*(*t*) = 0 coincides with the value of *T*_*c*_ at which the recall performance transfers from the primacy effect to the recency effect, as shown in Figures 2D, E. Specifically,

- When the maintenance period *T*_*maintain*_ is smaller than a critical value, i.e., $T_{c}^{\prime }\left(SIM\right)$ & *T*_*c*_, the recall performance exhibits the primacy effect; and the greater the value of Δ*Jux*(*T*_*maintain*_) is, the more significant the primacy effect becomes.
- As *T*_*maintain*_ approaches the critical value and Δ*Jux*(*T*_*maintain*_) approaches 0, the recall performance switches to the recency effect and is no longer significant (Figure 2D).
- When *T*_*maintain*_ is much larger than the critical value and meanwhile Δ*Jux*(*T*_*maintain*_)≫0, the recall performance displays the recency effect. The greater the value of Δ*Jux*(*T*_*maintain*_) is, the more pronounced the recency effect becomes.

It is worth noting that the temporal shift of the serial position effect is independent of the orientation difference between two visual stimuli, which is consistent with the results in Figures 2D, E. More details see Supplementary material and Supplementary Figure 3.

**Figure 3:**
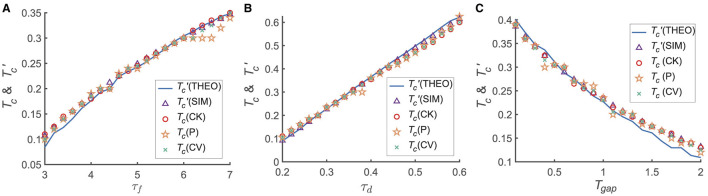
The dependence of *T*_*c*_, $T_{c}^{\prime }\left(THEO\&SIM\right)$ on different variables τ_*d*_, τ_*f*_ and *T*_*gap*_. The calculations for *T*_*c*_ employ various statistical methods, including normalized target probability (indicated by orange asterisks), Circular Variance (green crosses), and Circular Kurtosis (red hollow circles). The theoretical analyzes $T_{c}^{\prime }\left(THEO\right)$ represented by the blue solid line, while numerical simulations $T_{c}^{\prime }\left(SIM\right)$ denoted by purple triangles. **(A)** *T*_*c*_ and $T_{c}^{\prime }\left(THEO\&SIM\right)$ increase with increasing τ_*f*_; **(B)** *T*_*c*_ and $T_{c}^{\prime }\left(THEO\&SIM\right)$ increase with increasing τ_*d*_; **(C)** *T*_*c*_ and $T_{c}^{\prime }\left(THEO\&SIM\right)$ decrease with increasing *T*_*gap*_. $T_{c}^{\prime }\left(THEO\&SIM\right)\approx T_{c}$ in **(A–C)**. For each given parameter set { τ_*d*_, τ_*f*_, *T*_*gap*_ }, we computed the memory accuracy for different maintenance times *T*_*maintain*_ when the recall cue was presented. For every selected *T*_*maintain*_, the network was simulated 50 times, with each simulation comprising 300 trials. More details see Supplementary material 4.

We further investigated the detailed dependence of the transition from the primacy to the recency effect on the model and experimental parameters, including the time interval between two stimuli (*T*_*gap*_) and the parameters of STP (i.e., τ_*f*_ and τ_*d*_; Figure 3). For each set of parameters {τ_*f*_, τ_*d*_, *T*_*gap*_}, we calculated the memory accuracy of participants when the recall cue was given at different times (i.e., *T*_*maintain*_). For each given *T*_*maintain*_, we simulated the network 50 times, each consisting of 300 trails. We then calculated the transition moment from the primacy to the recency effect (i.e., *T*_*c*_) and the critical moment ($T_{c}^{\prime }\left(SIM\right)$) when Δ*Jux*(*t*)≡0. *T*_*c*_ is calculated using different statistical methods, such as CK, CV and P.

We found that both *T*_*c*_ and $T_{c}^{\prime }\left(SIM\right)$ increase with τ_*f*_ and τ_*d*_, respectively, as shown in Figures 3A, B, and they both decrease with *T*_*gap*_ (Figure 3C). Furthermore, *T*_*c*_ is approximately equal to $T_{c}^{\prime }\left(SIM\right)$ for each given parameter set, suggesting that the relative synaptic efficacy between two neural groups (*G*_1_ and *G*_2_) determines the recall performance. In conclusion, the shift of the serial position effect is determined by STP (i.e., τ_*f*_, τ_*d*_) and the inter-stimulus interval (*T*_*gap*_). Notably, as depicted in Figure 3, the transition from the primacy to the recency effect coincides with the time constant of STD (τ_*d*_), precisely aligning with its time order.

### 3.2 Theoretical analysis

In the above, we have utilized a simplified mean-field model to elucidate the neural mechanism of SWM, there are still many variables and parameters involved, including the time constants of STF and STD (τ_*f*_, τ_*d*_), the time constant of a single neuron (τ), the connection strength between neurons (i.e., *J*,*J*_0_,*J*_*EI*_,*J*_*IE*_, etc.), the duration of loading each stimulus (*T*_*encode*_), and the time interval between adjacent stimuli (*T*_*gap*_). If we continue using numerical simulations, it will be very time-consuming to explore how the recall performance depends on all these variables. We therefore conducted theoretical analysis to elucidate how the critical moment *T*_*c*_ depends on various variables that lead to the shift of the serial position effect. The advantage of theoretical analysis lies in its power of prediction, which can be validated with experiments. Specifically, we focused on examining the dependence of *T*_*c*_ on the variables τ_*f*_, τ_*d*_, and *T*_*gap*_.

To carry our theoretical analysis, we simplified the model of CANN with STP (i.e., Equations 1, 3, 5) into a model composed of multiple neuronal groups. In this simplified model, each *i*th neuronal group (*G*_*i*_, *i* = 1, ⋯ , *M* with *M* the number of items in the SWM task) encodes the *i*th item, and the interaction and overlap between different neuronal groups are ignored. This is because that the orientation difference between two stimuli does not impact much the recall performance, as shown in Supplementary Figure 3. Note that, the value of the visual orientation difference Δθ between memory items reflects the extent of interaction between neural groups encoding them. Using the experimental paradigm with two items as an example, we showed that the recall performances (Supplementary Figure 3) for Δθ being a random number in the range of [−π, +π] are consistent with that when Δθ takes a large value (Figures 2, 3, Δθ∈±17^*o*^, ±24^*o*^, ±38^*o*^, ±52^*o*^, ±66^*o*^, ±80^*o*^), indicating that the ignoring the interactions between neural groups is a proper approximation for theoretical analysis. Indeed, representation of too many items will introduce unignorable overlaps between neural groups. However, consider the limited capacity of working memory (~4 items, Zhang and Luck, 2008), ignoring the interactions between neuron groups is feasible in the theoretical analysis. The STP effect is considered in each neuronal group. Moreover, Figures 2, 3 have shown that the firing rates of different neuronal groups during the maintaining period approach zero (i.e., *r*_*i*_(*t*) → 0*Hz*), and this allows us to disregard the change in firing rates over time. On the other hand, the relative memory accuracy of participates for multiple items is mainly determined by the relative synaptic efficacy of neuronal groups during the maintaining period. Therefore, we only need to consider the dynamics of STP of different neuronal groups (*G*_*i*_) during the maintaining period, which is written as:


(6) $$\begin{array}{l} \frac{du_{i}\left(t\right)}{dt}=-\frac{u_{i}\left(t\right)}{\tau_{f}},\;\frac{dx_{i}\left(t\right)}{dt}=\frac{1-x_{i}}{\tau_{d}}, \end{array}$$

where *u*_*i*_(*t*) and *x*_*i*_(*t*) denote the STF and STD effects of the *i*th neuronal groups at time *t*, respectively, and *t* is aligned to the presence of the sencond item.

We first examined the case of two items (i.e., *M* = 2). In accordance with Equation (6), the synaptic efficacy of neuronal group *G*_*i*_ is calculated as $Jux_{i}\left(t\right)=Ju_{0}\exp\left(-\frac{t}{\tau_{f}}\right)\left[1-\left(1-x_{0}\right)\exp\left(-\frac{t}{\tau_{d}}\right)\right]$, where *u*_0_ and *x*_0_ represent the values of STF and STD of the *i*th neuronal group when the stimulus is removed. Thus, the relative synaptic efficacy among neuronal groups during the maintaining period is expressed as:


(7) $$\begin{array}{l} \;\Delta Jux(t)=Ju_{0}\exp\left(-\frac{t}{\tau_{f}}\right)\\ \left\{(1-x_{0})\exp\left(-\frac{t}{\tau_{d}}\right)\left[1-\exp\left(-\frac{t^{*}}{\tau_{d}}-\frac{t^{*}}{\tau_{f}}\right)\right]+\exp\left(-\frac{t^{*}}{\tau_{f}}\right)-1\right\}, \end{array}$$

where $t^{*}=T_{encode}+T_{gap}$. According to Equation (7), the critical moment $T_{c}^{{\;}^{\prime }}\left(THEO\right)$ for Δ*Jux*(*t*)≡0 is derived as,


(8) $$\begin{array}{l} T_{C}^{{\;}^{\prime }}\left(THEO\right)=\tau_{d}\ln\;\frac{\left(1-x_{0}\right)\left[1-\exp\left(-\frac{t^{*}}{\tau_{d}}-\frac{t^{*}}{\tau_{f}}\right)\right]}{1-\exp\left(-\frac{t^{*}}{\tau_{f}}\right)}. \end{array}$$

For details, see Supplementary material 3. Since it takes an amount of time for the cue in the recalling period to trigger the activity of the corresponding neuronal group, a constant bias (denoted as *t*_*b*_, *t*_*b*_∈(0, *T*_*recal*_]) is considered into Equation (8). Meanwhile, since τ_*f*_≫τ_*d*_, and $t^{*}=T_{encode}+T_{gap}$ has the same time scale as τ_*f*_, we further simplify Equation (8) to be:


(9) $$\begin{array}{l} T_{c}^{{\;}^{\prime }}\left(THEO\right)=\tau_{d}\ln\;\left[\frac{\left(1-x_{0}\right)}{1-\exp\left(-\frac{t^{*}}{\tau_{f}}\right)}\right]+t_{b}. \end{array}$$

Based on the theoretical predictions in Equation (9), we found that the theoretical results for $T_{c}^{\prime }\left(THEO\right)$ (represented by the blue colored line in Figure 3) are consistent with both the critical moment $T_{c}^{\prime }\left(SIM\right)$ (depicted by the purple triangle in Figure 3) and *T*_*c*_ (shown by the blue line in Figure 3). It is important to highlight that our theoretical analysis effectively captures the dynamic patterns of storage for multiple items qualitatively during the maintenance period. Therefore, the shift of the serial position effect is positively correlated with the time constants of STD and STF, which implies that a larger time constant of STD enables the working memory system to maintain the primacy effect for a longer duration. This shift of the serial position effect is inversely correlated with the sum of presentation durations of items and the inter-stimulus time intervals (referred to as t^*^), which indicates that the larger the value of t^*^, the more difficulty it is for the working memory system to keep the primacy effect.

**Figure 4:**
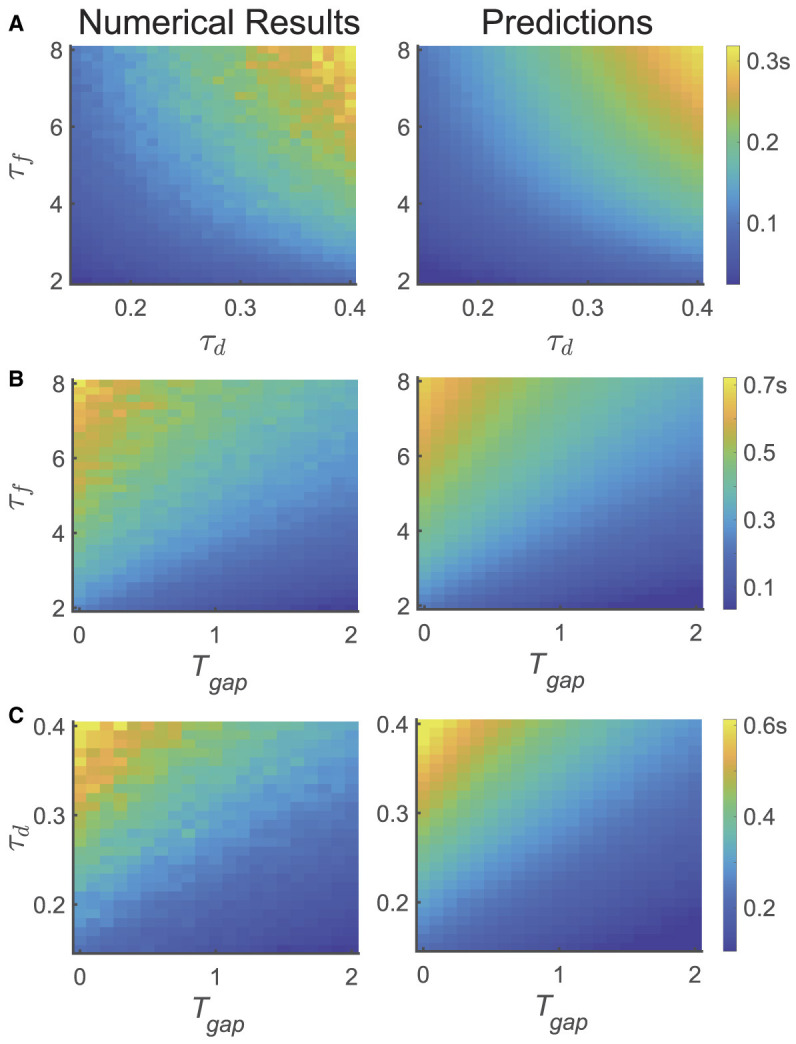
The dependence of temporal dynamics of memory accuracy on different variables and parameters, particularly τ_*f*_, τ_*d*_, and *T*_*gap*_. (left panel) the numerical simulation results of *T*_*c*_, (right panel) the theoretical predictions ($T_{c}^{{\;}^{\prime }}\left(THEO\right)$). **(A)** Both $T_{c}^{{\;}^{\prime }}\left(THEO\right)$ and *T*_*c*_ increases with increasing τ_*f*_, τ_*d*_. **(B)** Both $T_{c}^{{\;}^{\prime }}\left(THEO\right)$ and *T*_*c*_ increases with increasing τ_*f*_, and decreases with increasing *T*_*gap*_. **(C)** Both $T_{c}^{{\;}^{\prime }}\left(THEO\right)$ and *T*_*c*_ increases with increasing τ_*d*_, and decreases with increasing *T*_*gap*_.

To further validate the theoretical results, we conducted three different tasks to investigate the temporal dynamics of relative accuracy for multiple memory information (i.e., $T_{c}^{\prime }\left(THEO\right)$ and *T*_*c*_). We explored the serial position effect on various parameters of the network and the design of the experiment, as illustrated in Figure 4, where $T_{c}^{\prime }\left(THEO\right)$ is computed based on Equation (9).

In the first task, we calculated *T*_*c*_ using the same experimental parameters as depicted in Figure 2, but altered the values of τ_*f*_ and τ_*d*_ of the network, as shown in Figure 2A. We selected 21 different values of τ_*f*_ uniformly from the range of [2, 8] s and 21 different values of τ_*d*_ from the range of [0.1, 0.4] s. For each parameter set { τ_*f*_, τ_*d*_ }, we simulated the network and assessed recall performances of participants at different moments (i.e., *T*_*maintain*_), and obtained the critical moment *T*_*c*_ when the recall performance shifted from the primacy to the recency effect (left panel). The selection of *T*_*maintain*_ followed the same procedure as depicted Figure 3.

In the second task, we fixed τ_*d*_ and varied the variable *T*_*gap*_ in the psychophysical experiment, as well as τ_*f*_, as shown in Figure 4B. We selected 21 different values of τ_*f*_ uniformly from the range of [2, 8] s and 21 different values of *T*_*gap*_ from the range of [0, 2] s. The calculation method for *T*_*c*_ is the same as that in Figure 4A.

In the third task, we fixed τ_*f*_ and varied the variable *T*_*gap*_ in the psychophysical experiment, along with τ_*d*_, as shown in Figure 4C. We uniformly selected 21 different values of τ_*d*_ from the range of [0.1, 0.4] s and 21 different values of *T*_*gap*_ from the range of [0, 2] s. The calculation method for *T*_*c*_ is the same as in Figure 4A.

In summary, we found that: (1) the theoretical analysis of the critical moment ($T_{c}^{\prime }\left(THEO\right)$) is qualitatively consistent with the results obtained by numerical simulations (*T*_*c*_). (2) *T*_*c*_ increases with τ_*f*_ and τ_*d*_, as shown in Figure 4A, (3) *T*_*c*_ decreases with *T*_*gap*_, as shown in Figures 4B, C.

### 3.3 Model prediction: the serial position effect in SWM for multiple items

In this section, we demonstrate that the results obtained in SWM with two items can be extended to cases involving multiple items. We continued using the experimental paradigm depicted in Figure 2A for simulations, and studied the case of loading three items successively into the working memory system during the encoding period.

In the WM task, we considered that three items with different orientations are presented sequentially in each trail. Following a maintaining period lasting *T*_*maintain*_, a recalling signal with duration of *T*_*recall*_ is presented, which triggers the network to retrieve the task-related feature of the corresponding item. Denote *T*_*encode*_ the loading duration of each item, *T*_*gap*_ the time interval between two adjacent items, and θ_*i*_, (*i* = 1, 2, 3) the orientation of each item. The value of θ_*i*_(*i* = 1, 2, 3) is determined as follows. θ_1_ is randomly selected from the range of (−π/2, π/2], and θ_*i*_ is determined by θ_*i*_ = θ_1_+Δθ(*i* = 2, 3), where Δθ denotes the orientation difference between the *i*th (*i* = 2, 3) and the first items. The value of Δθ is chosen according to the relevant psychophysical experiments (Huang et al., 2023), specifically, Δθ∈[±12°, ±24°, ±36°, ±48°, ±60°, ±72°, ±84°], with Δθ being randomly selected in each trail. For each *T*_*maintain*_, we conducted 50 runs, each consisting of 500 trials, and used three methods of normalized target probability, Circular Variance, and Circular Kurtosis to measure the recall performance of each trail. More details are given in Supplementary material 1.2 and Supplementary Figure 2.

**Figure 5:**
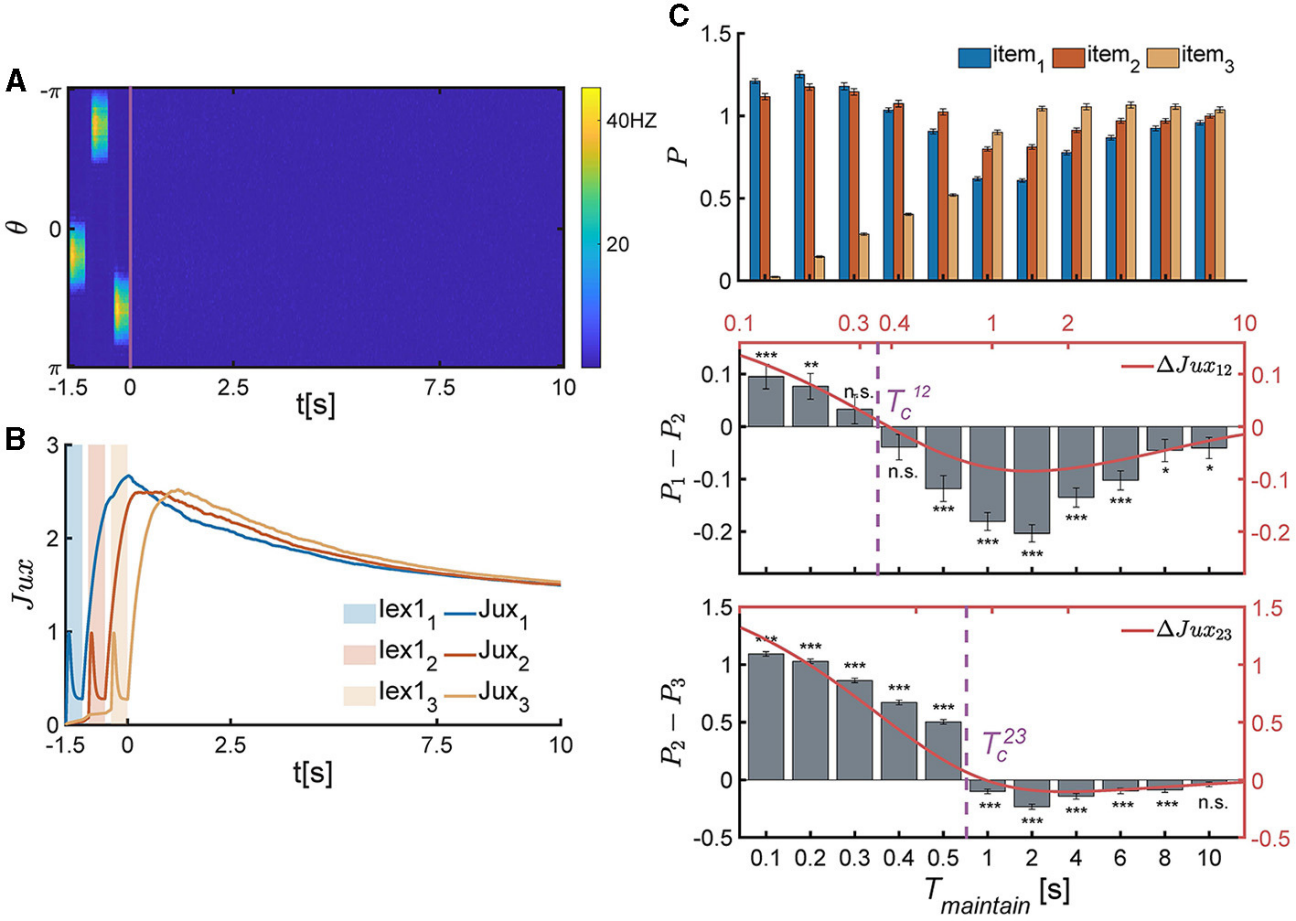
The temporal pattern of memory accuracy in a SWM task for three items. **(A, B)** The temporal dynamics of neural activity pattern in an individual trial simulation example. **(A)** Three items are presented sequentially loaded to the network, and the network generates three bump-shaped population firing pattern to represented the corresponding items, respectively. After the removal of items, the neural activity decays to a slient state. **(B)** The synaptic efficacy of each neuronal group (*Jux*_*i*_(*t*) for *i* = 1, 2, 3) rapidly decays when the corresponding items is presented, and then recovers to a maximum value, denoted as $Jux_{i}^{max}$, within a certain time and then remains at a high level for an extended duration. **(C)** The recall performance at varying *T*_*maintain*_. $T_{c}^{12}$ and $T_{c}^{23}$ present the critical moment of recall performance shift from the primacy to recency effects. (Top) The normalized target probability of the ith presented item, denoted as *P*_*i*_, *i* = 1, 2, 3, at varying *T*_*maintain*_. (Middle) The normalized target probability difference (denoted as *P*_1_−*P*_2_) and relative synaptic efficacy (*Jux*_1_−*Jux*_2_, the red line) between the 1st and 2nd items. (Bottom) The normalized target probability difference (denoted as *P*_2_−*P*_3_) and relative synaptic efficacy (*Jux*_2_−*Jux*_3_, the red line) between the 2nd and 3rd items. (n.s.: p >0.05, *: 0.01 <p <0.05, **: 0.001 <p <0.01, ***: p <0.001).

In each experimental trial, three items are sequentially loaded to the network, and the network generates three bump-shaped population firing patterns to represent the corresponding items (Figure 5A), respectively. The synaptic efficacy of each neuronal group (*Jux*_*i*_(*t*), *i* = 1, 2, 3) decreases rapidly due to the neurotransmitter depletion. After all items are removed, *Jux*_*i*_(*t*) (*i* = 1, 2, 3) recovers to its maximum value (Figure 5B) and then retains at a relatively high level to preserve the item information. We calculated the memory performance of participants at different *T*_*maintain*_, as shown in Figure 5C.

We extended the theoretical analysis in Section 4 to the SWM task involving three items. By comparing the relative synaptic efficacy of neuronal groups encoding different items, we can deduce the network's recalling performance. Due to the neglect of connections and overlaps between different neuronal groups, and the assumption that all neuronal groups receive the same intensity and duration of external signals, the relationship of synaptic efficiency between any two different neuronal groups (for example, *Jux*_*i*_(*t*), *Jux*_*j*_(*t*), and *i*<*j*, for the *i*th and *j* loaded items) during the maintaining period is solved to be $Jux_{i}\left(t\right)=Jux_{j}\left(t+\left(j-i\right)*t^{*}\right)$, with $t^{*}=T_{encode}+T_{gap}$. Thus, the relative synaptic efficacy between the *i*th and *j*th neuronal groups (Δ*Jux*_*ij*_(*t*)) are given by,


(10) $$\begin{array}{l} \;\Delta Jux_{12}(t)=\\ Ju_{0}\exp\left(\frac{t+t^{*}}{\tau_{f}}\right)\left\{(1-x_{0})\exp\left(-\frac{t+t^{*}}{\tau_{d}}\right)\left[1-\exp\left(-\frac{t^{*}}{\tau_{d}}-\frac{t^{*}}{\tau_{f}}\right)\right]+\exp\left(-\frac{t^{*}}{\tau_{f}}\right)-1\right\},\\ \;\Delta Jux_{23}(t)=\\ Ju_{0}\exp\left(\frac{t}{\tau_{f}}\right)\left\{(1-x_{0})\exp\left(-\frac{t}{\tau_{d}}\right)\left[1-\exp\left(-\frac{t^{*}}{\tau_{d}}-\frac{t^{*}}{\tau_{f}}\right)\right]+\exp\left(-\frac{t^{*}}{\tau_{f}}\right)-1\right\}. \end{array}$$

The critical moments (Equation 10) can be theoretically resolved based on Δ*Jux*_12_(*t*)≡0 and Δ*Jux*_23_(*t*)≡0, which are:


(11) $$\begin{array}{l} T_{c}^{12^{\prime }}=\tau_{d}\ln\left[\frac{1-x_{0}}{1-\exp(-\frac{t^{*}}{\tau_{f}})}-t^{*}+t_{b}^{12}\right],\\ T_{b}^{23^{\prime }}=\tau_{d}\ln\left[\frac{1-x_{0}}{1-\exp(-\frac{t^{*}}{\tau_{f}})}+t_{b}^{23}\right], \end{array}$$

where $t_{b}^{12},t_{b}^{23}$ $\left(t_{b}^{12},t_{b}^{23}\in \left(0,T_{recall}\right]\right)$ denote the response times of neuronal groups to recalling signals, which are approximately in the time order of τ. According to the above theoretical analysis, if $t<T_{c}^{12^{\prime }}$, then *Jux*_1_(*t*)>*Jux*_2_(*t*); otherwise, *Jux*_1_(*t*) <*Jux*_2_(*t*). Similarly, if $t<T_{c}^{23^{\prime }}$, then *Jux*_2_(*t*)>*Jux*_3_(*t*); otherwise, *Jux*_2_(*t*) <*Jux*_3_(*t*).

The above theoretical analysis is in perfect alignment with the numerical simulation results, as shown in Equation 11 and Figure 5C.

Firstly, when $T_{maintain}<T_{c}^{12^{\prime }}$, the network exhibits the primacy effect (see Figure 5C middle panel). This is because that after the removal of multiple items, *Jux*_1_ firstly recovers to the maximum valve, while the synaptic efficacy of other neuronal groups still remains low values due to neurotransmitter depletion (Figure 5B). The larger the relative synaptic efficacy Δ*Jux*_12_(*t*), the more significant the primacy effect. Meanwhile, both the value of Δ*Jux*_12_(*t*) and the significance of the primacy effect decrease with *T*_*maintain*_ over time. Thus, the recalling performance indeed shifts around the critical moment $T_{c}^{12^{\prime }}$ (Figure 5C).

Secondly, when $T_{maintain}>T_{c}^{23^{\prime }}$, the network exhibits the recency effect (see Figure 5C bottom panel). This is because the synaptic efficacy of the third item (*Jux*_3_(*t*)) recovers to its maximum valve, which is larger than that of other neuronal groups. Furthermore, the synaptic efficacy of neuronal groups gradually decrease over time, and the recency effect becomes insignificant (Figure 5C, bottom panel).

Thirdly, the critical moments ($T_{c}^{12^{\prime }}$ and $T_{c}^{23^{\prime }}$) are primarily influenced by the time constants of STF and STD (τ_*f*_ and τ_*d*_), as well as the time interval between adjacent items (*T*_*gap*_). Specifically, the values of $T_{c}^{12^{\prime }}$ and $T_{c}^{23^{\prime }}$ increase with τ_*f*_ and τ_*d*_, and decrease with *T*_*gap*_.

## 4 Discussion

In this study, we built a CANN incorporating STP to investigate the neural mechanism underlying the shift of the serial position effect in SWM. We found that with the elongation of the delay period, the participates' recall performance undergoes a shift from the pronounced primacy effect to the significant recency effect. Additionally, the prominence of the recency effect gradually wanes with the extension of the delay period. Furthermore, we show that the transition moment of the serial position effect is predominantly determined by STP, the time interval between adjacent stimuli, and the duration of stimulus presentation. We carried out theoretical analysis to confirm the simulation results and made predictions to be validated by future experiments. Overall, our study indicates that STP gives us insights into understanding how the ordinal information is processed in working memory.

Utilizing a CANN to depict the encoding, maintenance, and retrieval processes in SWM is biologically plausible. CANNs have been extensively applied to elucidate the neural mechanisms of information processing in working memory. In these tasks, the stimuli are typically represented by continuous variables' including visual orientation (Ben-Yishai et al., 1995), spatial location (Bottomley, 1987), and motion direction (Georgopoulos et al., 1986) in visual working memory, as well as auditory frequency in auditory working memory (Borderie et al., 2024). Additionally, CANNs have been widely used to describe the neural representation and storage of continuous variables, such as head orientation (Stringer et al., 2002; Wang and Kang, 2022), visual orientation (Li et al., 2021), motion direction (Fung et al., 2010; Mi et al., 2014), and spatial location (Samsonovich and McNaughton, 1997; Yoon et al., 2013), and they align with experimental data. A core feature of CANNs is that the synaptic connections between neurons are solely dependent on their difference in preferred continuous variables (Wu et al., 2013, 2016), implying that the synaptic connections in their feature space exhibit spatial translation invariance. Moreover, the network employs population coding (forming bump-shaped neural activity patterns) to represent external stimuli, where the peak value of the bump corresponds to the continuous value of the external stimulus. This structural characteristics of CANNs and its mode of representing external stimuli have been empirically validated.

In this study, we adopted the view of the synaptic mechanism of working memory which considers that information is stored in the facilitated synaptic connections (Mongillo et al., 2008). The prefrontal cortex (PFC) is a crucial cortical region for the execution of working memory. A significant body of empirical evidence demonstrates that the connections between neurons in the PFC exhibit STP and are dominated by STF (Wang et al., 2006; Masse et al., 2020; Bocincova et al., 2022). Consequently, after the removal of external stimuli, information can be stored in the temporally enhanced synaptic connections between neurons, rather than in the sustained firings of neurons (Rainer and Miller, 2002; Shafi et al., 2007), i.e., the memory is residing in the activity-silent hidden state.

The synaptic mechanism of working memory postulates that the memory information is primarily stored in the facilitated synaptic interactions between neurons, and the synaptic efficacy determines the accuracy of the memorized item (Stokes, 2015; Wolff et al., 2015). However, other studies suggest that STD also plays a critical role in the storage and manipulation processes of memory information. Regarding the storage of memory information, the limited capacity of working memory is directly proportional to the time constant of STD (Mi et al., 2017). In term of memory manipulation, for instance, external dynamic perturbations that are irrelevant to the task but weakly related to the attributes of the remembered items can alter the relative synaptic connection strengths between neuronal groups encoding different items through STD, thereby changing the relative accuracy of participates' recollection of different items in visual working memory in real time, a transition from the recency effect to the primacy effect (Knoedler et al., 1999; Li et al., 2021). Moreover, the time constant of STD determines the effective time window for dynamically manipulating working memory. Compared to previous works (particularly Li et al., 2021), the contributions of our study include: (1) We theoretically calculated the critical moment (*T*_*c*_) at which the relative memory accuracy of two or more items in a SWM task undergoes a transition. We showed that *T*_*c*_ depends on the time constants of short-term facilitation (τ_*f*_) and short-term depression (τ_*d*_), and the sum of the duration of presenting the items and the inter-item intervals (t^*^). (2) Previous studies focused on manipulating two memory items. In this study, we investigated the relative memory accuracy changes of multiple items and derived the critical moments for when these changes occur. Furthermore, our study indicates that the efficacy of synaptic connections (*Jux*) encoding memory items in neuron groups not only determines the accuracy of item retrieval but is also related to the ordinal information of items, suggesting the importance of STP in processing order information in SWM.

In our model, the efficacy of synaptic connections within neuronal groups, denoted as *Jux*, determines the accuracy of memory storage. On one hand, *u* represents the short-term synaptic facilitation effect. According to the synaptic computational theory of working memory, information is retained in the facilitated synaptic connections between neurons within each neuronal group. Therefore, the accuracy of information storage gradually diminishes as u decreases. On the other hand, *x* represents the short-term synaptic depression effect, which provides the network with a slow negative feedback effect. This negative feedback effect can induce the mobility of neuronal activity bumps within the network (Fung et al., 2012), consequently leading to the phenomenon where neural activity bumps drift in the feature space of the network during the information maintenance period [i.e., the memory drift phenomena reported by Funahashi et al. (1989)]. Such drifting of activity bumps weaken the accuracy of memory representation (Seeholzer et al., 2019). Therefore, during the information maintenance period, a decrease in *u* within the neuronal groups leads to memory accuracy decay; simultaneously, if the STD effect in the network is sufficiently strong, this effect can also induce the drifting of weak neuronal activities during the maintenance period, further reducing memory accuracy decay.

The design of the psychophysical experimental paradigm also affects the shift of the serial position effect. Our theoretical analysis and simulation results indicate that an increase in the duration of stimulus and the inter-stimulus interval can affect the relative synaptic efficacy between neuronal groups encoding different items. Consequently, this reduces the prominence of the primacy effect and its significance, and concurrently induces the shift of the serial position effect. These theoretical insights into the effects of the psychophysical experimental design on the serial position phenomena pave the way for further investigation into the control of SWM with multiple items.

## Data availability statement

The original contributions presented in the study are included in the article/Supplementary material, further inquiries can be directed to the corresponding author.

## Author contributions

JZ: Writing – original draft, Conceptualization, Formal analysis, Resources, Software, Validation, Visualization. LG: Writing – original draft, Data curation, Formal analysis, Methodology. XH: Writing – original draft. CM: Writing – original draft. YM: Conceptualization, Data curation, Formal analysis, Funding acquisition, Investigation, Methodology, Project administration, Resources, Software, Supervision, Validation, Visualization, Writing - original draft, Writing – review & editing.
