# Supplementary material for: The synaptic correlates of serial position effects in sequential working memory
Source: Front Comput Neurosci. 2024 Jul 15;18:1430244. doi: 10.3389/fncom.2024.1430244 (PMC11284078; doi:10.3389/fncom.2024.1430244)
Supplement: Supplementary file 1 [file Data_Sheet_1.PDF]

Supplementary Material

1 STATISTICAL METHODS

1.1 Population Vector Method

We used population vector method (Georgopoulos et al., 1982) to decode the orientation information encoded in neural activity in the network, which is given by:

$$\theta_{decode} = \frac{\int_{-\pi/2}^{\pi/2} \theta \langle r_E(\theta, t) \rangle d\theta}{\int_{-\pi/2}^{\pi/2} \langle r_E(\theta, t) \rangle d\theta}, \tag{S1}$$

where $\langle r_E(\theta, t) \rangle$ is the average firing rate of neurons at θ during the recalling period in each trail.

1.2 Normalized target probability and Circular variance, Circular kurtosis statistical method

1) Normalized target probability statistical method (Bays et al., 2009; Schneegans and Bays, 2016)

The normalized target probability method employs a three-component mixture model to describe the response probabilities of different orientations during the recalling period. The model comprises a Gaussian distribution centered around the target orientation, another Gaussian distribution centered around non-target orientations, and a uniform distribution representing random guessing. By fitting the model, we can determine the probability, denoted as p, of response based on the target orientation. Subsequently, p is normalized, resulting in the normalized target probability which serves as a metric for assessing recall performance.

2) Circular variance, Circular kurtosis statistical methods (Berens, 2009)

To evaluate the robustness of the statistical results, we also utilized Circular variance and Circular Kurtosis statistical methods to assess the recall performance. Circular variance is associated with the variance error distribution, whereas Circular Kurtosis represents the Kurtosis of the error distribution. A smaller Circular variance and a larger Circular Kurtosis indicate better recall performance. As illustrated in Figure S1, the participants' recall performance exhibits a transition from a primacy effect, similar to Figure 2D, to recency

effect, followed by a gradual decrease in the significance of the recency effect (Figure S1A (Paired t-test): $t_{49}^{0.1}(T_{maintain} = 0.1) = -16.146, p < 0.001; t_{49}^{0.2} = -9.393, p < 0.001; t_{49}^{0.3} = 0.158, p = 0.875; t_{49}^{0.4} = 5.618, p < 0.001; t_{49}^{0.5} = 10.287, p < 0.001; t_{49}^{1} = 14.424, p < 0.001; t_{49}^{2} = 13.358, p < 0.001; t_{49}^{4} = 6.398, p < 0.001; t_{49}^{6} = 5.384, p < 0.001; t_{49}^{8} = 1.901, p = 0.060; t_{49}^{10} = 1.640, p = 0.104$. Figure S1B (Paired t-test): $t_{49}^{0.1}(T_{maintain} = 0.1) = 17.396, p < 0.001; t_{49}^{0.2} = 8.743, p < 0.001; t_{49}^{0.3} = -0.430, p = 0.668; t_{49}^{0.4} = -3.956, p < 0.001; t_{49}^{0.5} = -10.104, p < 0.001; t_{49}^{1} = -13.833, p < 0.001; t_{49}^{2} = -13.695, p < 0.001; t_{49}^{4} = -5.639, p < 0.001; t_{49}^{6} = -4.512, p < 0.001; t_{49}^{8} = -1.302, p = 0.196; t_{49}^{10} = -0.918, p = 0.361$) . Additionally, the recall performance also shows a a similar trend as in Figure 2D in the Maintext. In the 3-items task, as shown in FigureS2, the recall effects of the participants followed a similar trend as in Figure 5BC. Specifically, when $T_{maintain}$ is less than $T_c^{12'}$, participants show a primacy effect, and when $T_{maintain} > T_c^{23'}$, the recall performance shifts to a recency effect (Figure S2A, the middle (Paired t-test): $t_{49}^{0.1}(T_{maintain} = 0.1) = -10.566, p < 0.001; t_{49}^{0.2} = -4.405, p < 0.001; t_{49}^{0.3} = 0.460, p = 0.647; t_{49}^{0.4} = 1.926, p = 0.057; t_{49}^{0.5} = 5.244, p < 0.001; t_{49}^{1} = 8.331, p < 0.001; t_{49}^{2} = 9.976, p < 0.001; t_{49}^{4} = 6.114, p < 0.001; t_{49}^{6} = 5.207, p < 0.001; t_{49}^{8} = 2.708, p = 0.008; t_{49}^{10} = 2.711, p = 0.008$; the bottom: $t_{49}^{0.1}(T_{maintain} = 0.1) = -110.634, p < 0.001; t_{49}^{0.2} = -76.014, p < 0.001; t_{49}^{0.3} = -54.249, p < 0.001; t_{49}^{0.4} = -43.133, p < 0.001; t_{49}^{0.5} = -31.812, p < 0.001; t_{49}^{1} = 5.458, p < 0.001; t_{49}^{2} = 13.697, p < 0.001; t_{49}^{4} = 8.937, p < 0.001; t_{49}^{6} = 4.857, p < 0.001; t_{49}^{8} = 4.042, p < 0.001; t_{49}^{10} = 1.854, p = 0.067$. Figure S2B, the middle (Paired t-test): $t_{49}^{0.1}(T_{maintain} = 0.1) = 12.046, p < 0.001; t_{49}^{0.2} = 4.091, p < 0.001; t_{49}^{0.3} = -2.369, p = 0.020; t_{49}^{0.4} = -5.069, p < 0.001; t_{49}^{0.5} = -7.535, p < 0.001; t_{49}^{1} = -14.287, p < 0.001; t_{49}^{2} = -12.406, p < 0.001; t_{49}^{4} = -6.094, p < 0.001; t_{49}^{6} = -4.798, p < 0.001; t_{49}^{8} = -2.423, p = 0.017; t_{49}^{10} = -1.685, p = 0.095$; the bottom: $t_{49}^{0.1}(T_{maintain} = 0.1) = 33.897, p < 0.001; t_{49}^{0.2} = 43.528, p < 0.001; t_{49}^{0.3} = 37.554, p < 0.001; t_{49}^{0.4} = 25.426, p < 0.001; t_{49}^{0.5} = 20.016, p < 0.001; t_{49}^{1} = -2.933, p < 0.001; t_{49}^{2} = -11.441, p < 0.001; t_{49}^{4} = -8.322, p < 0.001; t_{49}^{6} = -3.341, p = 0.001; t_{49}^{8} = -3.018, p = 0.003; t_{49}^{10} = -1.447, p = 0.151$).

For each maintaining duration, we calculated the recall performance of ith item ($\langle\mu\rangle_i^s(METH)$) using three statistical methods for the trials in which the ith is recalled. The recall performance was then averaged across all runs to obtain the final recalling performance ($\langle\mu\rangle_i(METH)$) at different $T_{miantain}$.

$$\langle\mu\rangle_i(METH) = \frac{1}{50}\sum_{s=1}^{50}\langle\mu\rangle_i^s(METH), \tag{S2}$$

where $\mu_i^s(METH)$ denotes the recalling performance of the ith item using the METH statistical method (where METH=Normalized Target Probability, Circular variance, Circular kurtosis) in the sth run.

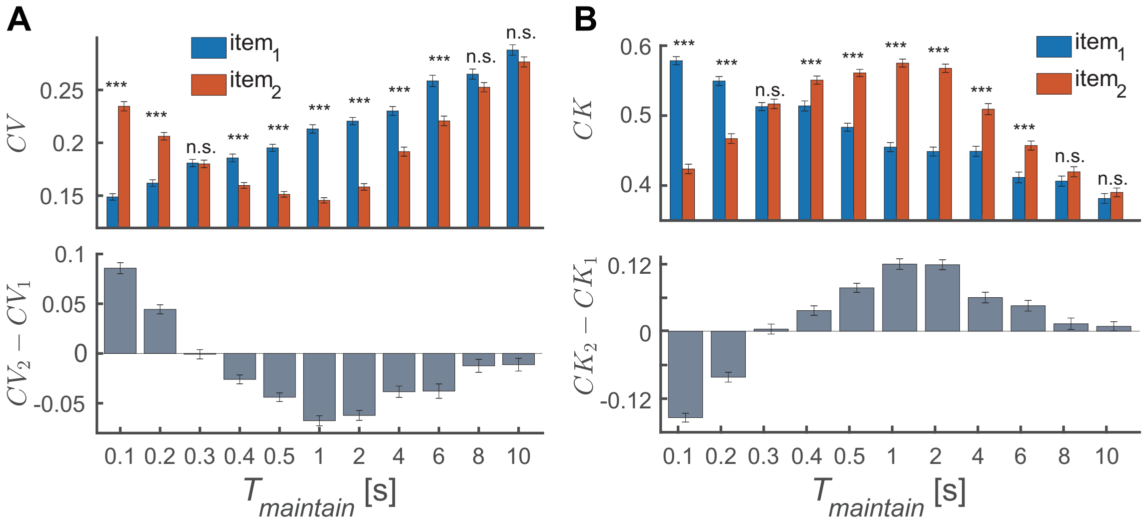

Figure S1. The recall performance at varyting $T_{maintain}$. (A) (Top)The recall performance of the ith presented item, denotes as CV_i for $i = 1, 2$, calculated using Circular variance method. (Bottom) The recall performance difference, denoted as $CV_1 - CV_2$, between the 1st and 2nd items. (B) (Top) The recall performance of the ith presented item, denotes as CK_i for $i = 1, 2$, calculated using Circular variance method. (Bottom) The recall performance difference, denoted as $CK_1 - CK_2$, between the 1st and 2nd items. (n.s.: p >0.05, $***$: p <0.001)

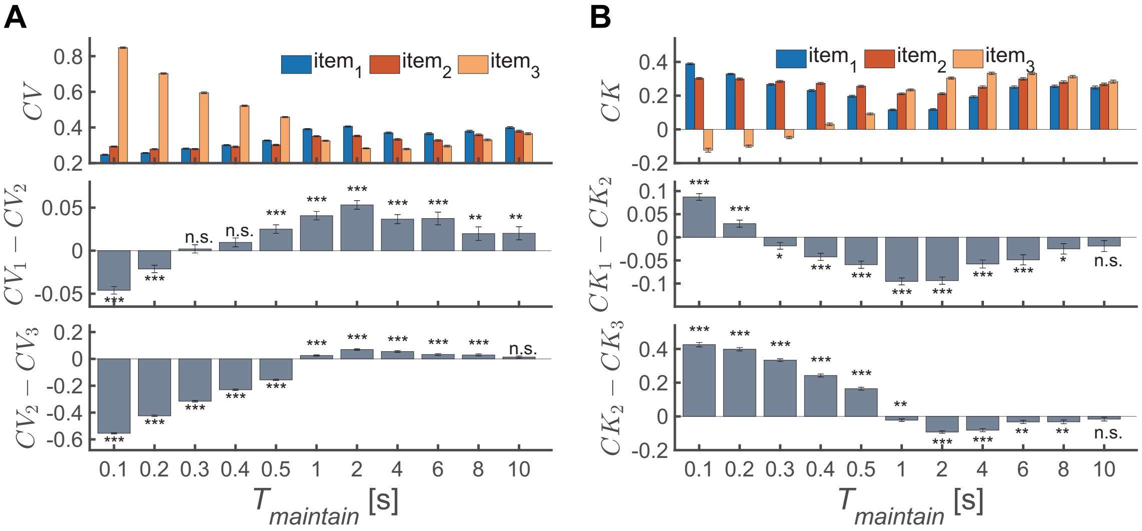

Figure S2. The recall performance at varying $T_{maintain}$. (A) (Top)The recall performance of the ith presented item, denotes as CV_i for $i = 1, 2, 3$, calculated using Circular variance method. (Middle)(Bottom) The recall performance difference $CV_1 - CV_2$ and $CV_2 - CV_3$. (B) (Top) The recall performance of the ith presented item, denotes as CK_i for $i = 1, 2, 3$, calculated using Circular variance method. (Middle)(Bottom) The recall performance difference $CK_1 - CK_2$ and $CK_2 - CK_3$. (n.s.: p >0.05, $*$: 0.01 <p <0.05, $**$: 0.001 <p <0.01, $***$: p <0.001)

2 RECALL PERFORMANCE UNDER THE CONDITION OF RANDOM RELATIVE ORIENTATION DIFFERENCES

To investigate whether the recall performance is affected by the relative angle difference between two items (i.e., $\Delta\theta$), we randomly selected the relative difference $\Delta\theta$ in the range of $[-\pi, +\pi]$ and observed similar results to Figure 2D (depicted in Figure S3), which are: A (paired t-test): $t^{0.1}_{49}(T_{maintain} = 0.1) = 5.073, p < 0.001; t^{0.2}_{49} = 4.017, p < 0.001; t^{0.3}_{49} = 1.065, p = 0.289; t^{0.4}_{49} = -0.177, p = 0.860; t^{0.5}_{49} = -4.286, p < 0.001; t^{1}_{49} = -7.689, p < 0.001; t^{2}_{49} = -6.536, p < 0.001; t^{4}_{49} = -0.589, p = 0.557; t^{6}_{49} = -1.922, p = 0.058; t^{8}_{49} = 0.199, p = 0.843; t^{10}_{49} = -0.180, p = 0.858$. B (Paired t-test): $t^{0.1}_{49}(T_{maintain} = 0.1) = -10.821, p < 0.001; t^{0.2}_{49} = -6.021, p < 0.001; t^{0.3}_{49} = -1.261, p = 0.210; t^{0.4}_{49} = 4.096, p < 0.001; t^{0.5}_{49} = 5.851, p < 0.001; t^{1}_{49} = 11.588, p < 0.001; t^{2}_{49} = 11.478, p < 0.001; t^{4}_{49} = 6.208, p < 0.001; t^{6}_{49} = 5.881, p < 0.001; t^{8}_{49} = 0.265, p = 0.791; t^{10}_{49} = 1.008, p = 0.316$. C (Paired t-test): $t^{0.1}_{49}(T_{maintain} = 0.1) = 9.158, p < 0.001; t^{0.2}_{49} = 5.079, p < 0.001; t^{0.3}_{49} = 0.159, p = 0.874; t^{0.4}_{49} = -4.033, p < 0.001; t^{0.5}_{49} = -4.067, p < 0.001; t^{1}_{49} = -8.507, p < 0.001; t^{2}_{49} =$

$-9.355, p < 0.001; t^4_{49} = -7.091, p < 0.001; t^6_{49} = -5.730, p < 0.001; t^8_{49} = -1.456, p = 0.149; t^{10}_{49} = -0.334, p = 0.739$). Specifically, when $T_{maintain} < T_c$, the recall performance shows a primacy effect. As $T_{maintain}$ approches T_c, the significance of primacy effect decreases. When $T_{maintain} > T_c$, the recall performance switches to a recency effect, with the significance gradually decreasing. Additionally, T_c is approximately equal to the critical moment when $\Delta Jux(t) \equiv 0$.

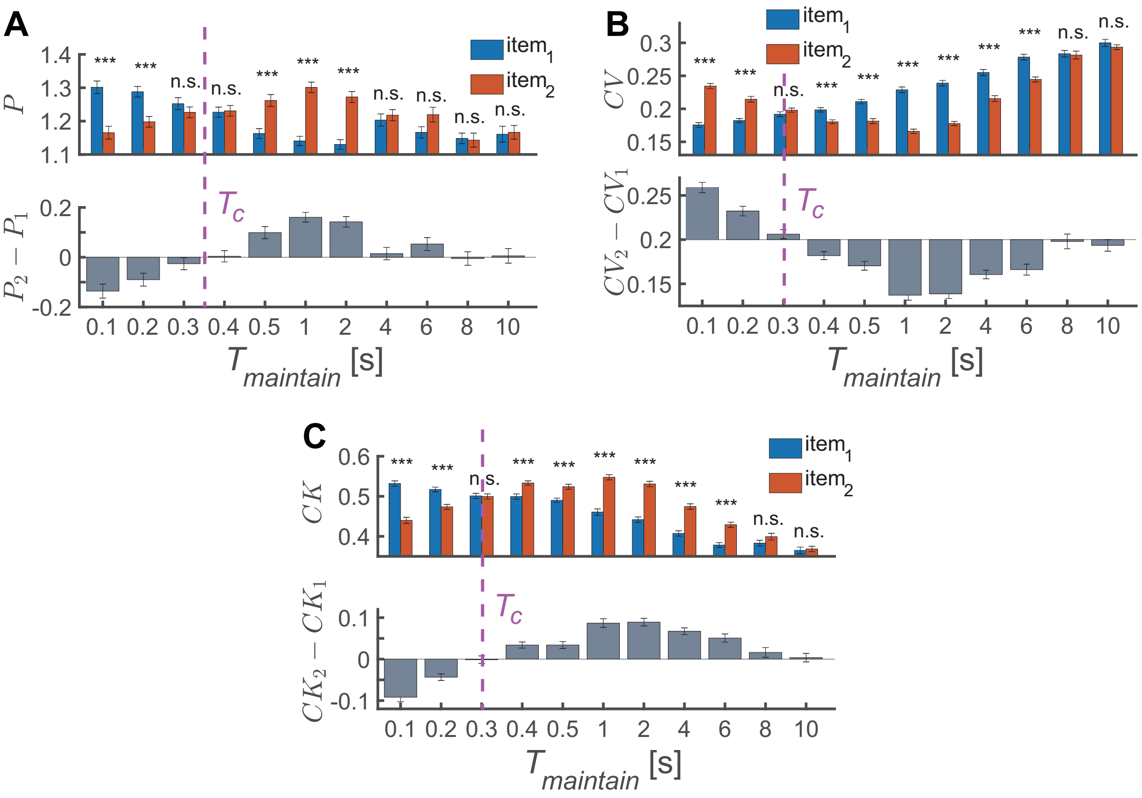

Figure S3. The recall performance at varying $T_{maintain}$, where both orientated value of items are selected randomly. The recall performance are calculated using different statical methods, including the normalized target probability (A) and Circular variance (B), Circular kurtosis methods (C). T_c is the critical moment of recall performance shift from primacy to recency effect. (n.s.: p >0.05, $***$: p <0.001)

3 THEORETICAL ANALYSIS

3.1 The mathematical expression of $Jux_i(t)$

1) The mathematical expression of the release probability of neurotransmitters over time

According to Eq.6 in the Maintext, (i.e., $\frac{du_i(t)}{dt} = -\frac{u_i(t)}{\tau_f}$),

$$\frac{du_i}{u_i} = -\frac{dt}{\tau_f} \Rightarrow \ln u_i = -\frac{t}{\tau_f} + C_1 \Rightarrow u_i = \exp\left(-\frac{t}{\tau_f} + C_1\right) = u_0 \exp\left(-\frac{t}{\tau_f}\right), \quad \text{(S3)}$$

where u_0 denotes the value of $u_i(t)$ at the moment of the ith item removes.

2) The mathematical expression of the proportion of available neurotransmitters over time

According to Eq.6 in the Maintext, (i.e., $\frac{dx_i}{dt} = \frac{(1-x_i)}{\tau_d}$), we defines $\widetilde{x}_i = 1 - x_i$, which is given by

$$\frac{d\widetilde{x}_i}{dt} = \frac{\widetilde{x}_i}{\tau_d} \Rightarrow \ln \widetilde{x}_i = -\frac{t}{\tau_d} + C_2 \Rightarrow \widetilde{x}_i(\theta, t) = \exp\left(-\frac{t}{\tau_d} + C_2\right),$$

and thus,

$$x_i = 1 - \exp\left(-\frac{t}{\tau_d} + C_2\right) = 1 - (1 - x_0)\exp\left(-\frac{t}{\tau_d}\right), \quad \text{(S4)}$$

where x_0 is the value of $x_i(t)$ at the moment of the ith item removes.

Thus, the synaptic efficacy of ith neuronal group is given by

$$Jux_i(t) = Ju_i(\theta, t)x_i(\theta, t) = Ju_0 \exp\left(-\frac{t}{\tau_f}\right)[1 - (1 - x_0)\exp\left(-\frac{t}{\tau_d}\right)], \quad \text{(S5)}$$

3.2 The mathematical expression of the relative synaptic efficacy between neuronal groups over time (Eq.9)

According to the psychophysical experimental paradigm, we define $t^* = T_{encode} + T_{gap}$, and the relative synaptic efficacy between neuronal groups in the maintaining period is

given by

$$\Delta Jux(t) = Jux_1(t+t^*) - Jux_2(t)$$
$$= Ju_0 \exp\left(-\frac{t+t^*}{\tau_f}\right)\left[1-(1-x_0)\exp\left(-\frac{t+t^*}{\tau_d}\right)\right] -$$
$$Ju_0 \exp\left(-\frac{t}{\tau_f}\right)\left[1-(1-x_0)\exp\left(-\frac{t}{\tau_d}\right)\right]$$
$$= Ju_0 \exp\left(-\frac{t}{\tau_f}\right)\left\{(1-x_0)\exp\left(-\frac{t}{\tau_d}\right)\left[1-\exp\left(-\frac{t^*}{\tau_d}-\frac{t^*}{\tau_f}\right)\right]+\exp\left(-\frac{t^*}{\tau_f}\right)-1\right\}, \tag{S6}$$

By solving the equation $\Delta Jux(t) \equiv 0$, the critical moment $T_c^{'}$ is calculated by,

$$T_c^{'}(THEO) = \tau_d \ln \frac{(1-x_0)\left[1-\exp\left(-\frac{t^*}{\tau_d}-\frac{t^*}{\tau_f}\right)\right]}{1-\exp\left(-\frac{t^*}{\tau_f}\right)} = \tau_d \ln\left[(1-x_0)\frac{1-\exp\left(-\frac{t^*}{\tau_d}\left(1+\frac{\tau_d}{\tau_f}\right)\right)}{1-\exp\left(-\frac{t^*}{\tau_f}\right)}\right],$$

Since $\tau_d \ll \tau_f, t^*$, the critical moment is further simplified,

$$T_c^{'}(THEO) = \tau_d \ln\left[\frac{(1-x_0)}{1-\exp(\frac{t^*}{\tau_f})}\right] + t_b,$$

4 THE PARAMETERS

The parameters used in the above simulations are as follows S1, S2.

Table S1. Parameters in 2-items task

The CANN Parameters		
N	Number of neurons in the CANN	100
J	Parameter controlling the neuronal interactions strength in the CANN	4
J_0	Parameter controlling the baseline of neuronal interactions strength in the CANN	0.9
B	Parameter controlling the range of neuronal interactions in the CANN	4
J_{IE}	Connection strength from neurons in the CANN to inhibitory pool	1.1
J_{EI}	Connection strength from inhibitory pool to neurons in the CANN	0.13
τ	Time constant of synaptic current of neurons	0.01s
α	Parameter controlling the value of r_E near $h_E \approx 0$	1.5
I_0	Strength of background current input in CANN	-2.8
σ_0	Strength of background input noise	1.9
The STP Parameters		
τ_f	Time constant of STF	6.1s
τ_d	Time constant of STD	0.36s
U_0	Increment of utilization ratio of neurotransmitter	0.15
Parameters of Visual Stimulus Signals		
a_{encode}	Strength of color bar stimulus	13Hz
B_{encode}	Parameter controlling the accuracy of color bar stimulus	4
σ_{encode}	Strength of the noise of color bar stimulus	1
T_{encode}	Duration of color bar stimulus	1s
T_{gap}	Time gap between two color bar stimuli	0.5s
Parameters of Recalling Signals		
a_{recall}	Strength of recall signal	0.145
B_{recall}	Parameter controlling the accuracy of recall signal	1.8
σ_{recall}	Strength of the noise of recall signal	0.12
T_{recall}	Duration of recall signal	0.24s
Parameters of theoretical analysis		
t_b	Constant bias	0.16s

Table S2. Parameters in 3-items task

The CANN Parameters		
N	Number of neurons in the CANN	100
J	Parameter controlling the neuronal interactions strength in the CANN	4
J_0	Parameter controlling the baseline of neuronal interactions strength in the CANN	0.01
B	Parameter controlling the range of neuronal interactions in the CANN	3.6
J_{IE}	Connection strength from neurons in the CANN to inhibitory pool	1.1
J_{EI}	Connection strength from inhibitory pool to neurons in the CANN	0.15
τ	Time constant of synaptic current of neurons	0.01s
α	Parameter controlling the value of r_E near $h_E \approx 0$	1.5
I_0	Strength of background current input in CANN	-2
σ_0	Strength of background input noise	1.7
The STP Parameters		
τ_f	Time constant of STF	6.1s
τ_d	Time constant of STD	0.47s
U_0	Increment of utilization ratio of neurotransmitter	0.22
Parameters of Visual Stimulus Signals		
a_{encode}	Strength of color bar stimulus	30Hz
B_{encode}	Parameter controlling the accuracy of color bar stimulus	3.6
σ_{encode}	Strength of the noise of color bar stimulus	1
T_{encode}	Duration of color bar stimulus	0.4s
T_{gap}	Time gap between two color bar stimuli	0.15s
Parameters of Recalling Signals		
a_{recall}	Strength of recall signal	0.16
B_{recall}	Parameter controlling the accuracy of recall signal	1
σ_{recall}	Strength of the noise of recall signal	0.08
T_{recall}	Duration of recall signal	0.13s
Parameters of theoretical analysis		
t_b	Constant bias	0.16s

5 STATISTICAL HYPOTHESIS TESTING

Table S3. The paired t-test for P1 and P2 under different T_{maintain} in two-item memory task

T_{maintain}	t-value	p-value
0.1	$t_{49}^{0.1} = 9.428$	$p < 0.001$
0.2	$t_{49}^{0.2} = 5.259$	$p < 0.001$
0.3	$t_{49}^{0.3} = -0.574$	$p = 0.568$
0.4	$t_{49}^{0.4} = -2.739$	$p = 0.007$
0.5	$t_{49}^{0.5} = -5.683$	$p < 0.001$
1	$t_{49}^{1} = -7.327$	$p < 0.001$
2	$t_{49}^{2} = -6.310$	$p < 0.001$
4	$t_{49}^{4} = -5.613$	$p < 0.001$
6	$t_{49}^{6} = -3.124$	$p = 0.002$
8	$t_{49}^{8} = -2.499$	$p = 0.014$
10	$t_{49}^{10} = -1.309$	$p = 0.194$

Table S4. The paired t-test for P1 and P2 under different T_{maintain} in three-item memory task

T_{maintain}	t-value	p-value
0.1	$t_{49}^{0.1} = 4.086$	$p < 0.001$
0.2	$t_{49}^{0.2} = 2.737$	$p = 0.007$
0.3	$t_{49}^{0.3} = 1.152$	$p = 0.252$
0.4	$t_{49}^{0.4} = -1.599$	$p = 0.113$
0.5	$t_{49}^{0.5} = -4.893$	$p < 0.001$
1	$t_{49}^{1} = -10.311$	$p < 0.001$
2	$t_{49}^{2} = -11.748$	$p < 0.001$
4	$t_{49}^{4} = -6.886$	$p < 0.001$
6	$t_{49}^{6} = -5.163$	$p < 0.001$
8	$t_{49}^{8} = -2.264$	$p = 0.026$
10	$t_{49}^{10} = -2.162$	$p = 0.033$

Table S5. The paired t-test for P2 and P3 under different T_{maintain} in three-item memory task

$T_{\textbf{maintain}}$	**t-value**	**p-value**
0.1	$t_{49}^{0.1} = 57.384$	$p < 0.001$
0.2	$t_{49}^{0.2} = 50.442$	$p < 0.001$
0.3	$t_{49}^{0.3} = 41.880$	$p < 0.001$
0.4	$t_{49}^{0.4} = 31.587$	$p < 0.001$
0.5	$t_{49}^{0.5} = 24.866$	$p < 0.001$
1	$t_{49}^{1} = -5.331$	$p < 0.001$
2	$t_{49}^{2} = -11.678$	$p < 0.001$
4	$t_{49}^{4} = -6.059$	$p < 0.001$
6	$t_{49}^{6} = -4.163$	$p < 0.001$
8	$t_{49}^{8} = -4.105$	$p < 0.001$
10	$t_{49}^{10} = -1.689$	$p < 0.001$

Table S6. The paired t-test for CV1 and CV2 under different T_{maintain} in two-item memory task

$T_{\textbf{maintain}}$	**t-value**	**p-value**
0.1	$t_{49}^{0.1} = -16.146$	$p < 0.001$
0.2	$t_{49}^{0.2} = -9.393$	$p < 0.001$
0.3	$t_{49}^{0.3} = 0.158$	$p = 0.875$
0.4	$t_{49}^{0.4} = 5.618$	$p < 0.001$
0.5	$t_{49}^{0.5} = 10.287$	$p < 0.001$
1	$t_{49}^{1} = 14.424$	$p < 0.001$
2	$t_{49}^{2} = 13.358$	$p < 0.001$
4	$t_{49}^{4} = 6.398$	$p < 0.001$
6	$t_{49}^{6} = 5.384$	$p < 0.001$
8	$t_{49}^{8} = 1.901$	$p = 0.060$
10	$t_{49}^{10} = 1.640$	$p = 0.104$

Table S7. The paired t-test for CK1 and CK2 under different T_{maintain} in two-item memory task

$T_{\textbf{maintain}}$	**t-value**	**p-value**
0.1	$t_{49}^{0.1} = 17.396$	$p < 0.001$
0.2	$t_{49}^{0.2} = 8.743$	$p < 0.001$
0.3	$t_{49}^{0.3} = -0.430$	$p = 0.668$
0.4	$t_{49}^{0.4} = -3.956$	$p < 0.001$
0.5	$t_{49}^{0.5} = -10.104$	$p < 0.001$
1	$t_{49}^{1} = -13.833$	$p < 0.001$
2	$t_{49}^{2} = -13.695$	$p < 0.001$
4	$t_{49}^{4} = -5.639$	$p < 0.001$
6	$t_{49}^{6} = -4.512$	$p < 0.001$
8	$t_{49}^{8} = -1.302$	$p = 0.196$
10	$t_{49}^{10} = -0.918$	$p = 0.361$

Table S8. The paired t-test for CV1 and CV2 under different T_{maintain} in three-item memory task

$T_{\textbf{maintain}}$	**t-value**	**p-value**
0.1	$t_{49}^{0.1} = -10.566$	$p < 0.001$
0.2	$t_{49}^{0.2} = -4.405$	$p < 0.001$
0.3	$t_{49}^{0.3} = 0.460$	$p = 0.647$
0.4	$t_{49}^{0.4} = 1.926$	$p = 0.057$
0.5	$t_{49}^{0.5} = 5.244$	$p < 0.001$
1	$t_{49}^{1} = 8.331$	$p < 0.001$
2	$t_{49}^{2} = 9.976$	$p < 0.001$
4	$t_{49}^{4} = 6.114$	$p < 0.001$
6	$t_{49}^{6} = 5.207$	$p < 0.001$
8	$t_{49}^{8} = 2.708$	$p = 0.008$
10	$t_{49}^{10} = 2.711$	$p = 0.008$

Table S9. The paired t-test for CV2 and CV3 under different T_{maintain} in three-item memory task

$T_{\textbf{maintain}}$	**t-value**	**p-value**
0.1	$t_{49}^{0.1} = -110.634$	$p < 0.001$
0.2	$t_{49}^{0.2} = -76.014$	$p < 0.001$
0.3	$t_{49}^{0.3} = -54.249$	$p < 0.001$
0.4	$t_{49}^{0.4} = -43.133$	$p < 0.001$
0.5	$t_{49}^{0.5} = -31.812$	$p < 0.001$
1	$t_{49}^{1} = 5.458$	$p < 0.001$
2	$t_{49}^{2} = 13.697$	$p < 0.001$
4	$t_{49}^{4} = 8.937$	$p < 0.001$
6	$t_{49}^{6} = 4.857$	$p < 0.001$
8	$t_{49}^{8} = 4.042$	$p < 0.001$
10	$t_{49}^{10} = 1.854$	$p = 0.067$

Table S10. The paired t-test for CK1 and CK2 under different T_{maintain} in three-item memory task

$T_{\textbf{maintain}}$	**t-value**	**p-value**
0.1	$t_{49}^{0.1} = 12.046$	$p < 0.001$
0.2	$t_{49}^{0.2} = 4.091$	$p < 0.001$
0.3	$t_{49}^{0.3} = -2.369$	$p = 0.020$
0.4	$t_{49}^{0.4} = -5.069$	$p < 0.001$
0.5	$t_{49}^{0.5} = -7.535$	$p < 0.001$
1	$t_{49}^{1} = -14.287$	$p < 0.001$
2	$t_{49}^{2} = -12.406$	$p < 0.001$
4	$t_{49}^{4} = -6.094$	$p < 0.001$
6	$t_{49}^{6} = -4.798$	$p < 0.001$
8	$t_{49}^{8} = -2.423$	$p = 0.017$
10	$t_{49}^{10} = -1.685$	$p = 0.095$

Table S11. The paired t-test for CK2 and CK3 under different T_{maintain} in three-item memory task

T_{maintain}	t-value	p-value
0.1	$t_{49}^{0.1} = 33.897$	$p < 0.001$
0.2	$t_{49}^{0.2} = 43.528$	$p < 0.001$
0.3	$t_{49}^{0.3} = 37.554$	$p < 0.001$
0.4	$t_{49}^{0.4} = 25.426$	$p < 0.001$
0.5	$t_{49}^{0.5} = 20.016$	$p < 0.001$
1	$t_{49}^{1} = -2.933$	$p < 0.001$
2	$t_{49}^{2} = -11.441$	$p < 0.001$
4	$t_{49}^{4} = -8.322$	$p < 0.001$
6	$t_{49}^{6} = -3.341$	$p = 0.001$
8	$t_{49}^{8} = -3.018$	$p = 0.003$
10	$t_{49}^{10} = -1.447$	$p = 0.151$

Table S12. The paired t-test for P1 and P2 under different T_{maintain} in two-item memory task under the condition of random relative angle differences

T_{maintain}	t-value	p-value
0.1	$t_{49}^{0.1} = 5.073$	$p < 0.001$
0.2	$t_{49}^{0.2} = 4.017$	$p < 0.001$
0.3	$t_{49}^{0.3} = 1.065$	$p = 0.289$
0.4	$t_{49}^{0.4} = -0.177$	$p = 0.860$
0.5	$t_{49}^{0.5} = -4.286$	$p < 0.001$
1	$t_{49}^{1} = -7.689$	$p < 0.001$
2	$t_{49}^{2} = -6.536$	$p < 0.001$
4	$t_{49}^{4} = -0.589$	$p = 0.557$
6	$t_{49}^{6} = -1.922$	$p = 0.058$
8	$t_{49}^{8} = 0.199$	$p = 0.843$
10	$t_{49}^{10} = -0.180$	$p = 0.858$

Table S13. The paired t-test for CV1 and CV2 under different T_{maintain} in two-item memory task under the condition of random relative angle differences

T_{maintain}	**t-value**	**p-value**
0.1	$t_{49}^{0.1} = -10.821$	$p < 0.001$
0.2	$t_{49}^{0.2} = -6.021$	$p < 0.001$
0.3	$t_{49}^{0.3} = -1.261$	$p = 0.210$
0.4	$t_{49}^{0.4} = 4.096$	$p < 0.001$
0.5	$t_{49}^{0.5} = 5.851$	$p < 0.001$
1	$t_{49}^{1} = 11.588$	$p < 0.001$
2	$t_{49}^{2} = 11.478$	$p < 0.001$
4	$t_{49}^{4} = 6.208$	$p < 0.001$
6	$t_{49}^{6} = 5.881$	$p < 0.001$
8	$t_{49}^{8} = 0.265$	$p = 0.791$
10	$t_{49}^{10} = 1.008$	$p = 0.316$

Table S14. The paired t-test for CK1 and CK2 under different T_{maintain} in two-item memory task under the condition of random relative angle differences

T_{maintain}	**t-value**	**p-value**
0.1	$t_{49}^{0.1} = 9.158$	$p < 0.001$
0.2	$t_{49}^{0.2} = 5.079$	$p < 0.001$
0.3	$t_{49}^{0.3} = 0.159$	$p = 0.874$
0.4	$t_{49}^{0.4} = -4.033$	$p < 0.001$
0.5	$t_{49}^{0.5} = -4.067$	$p < 0.001$
1	$t_{49}^{1} = -8.507$	$p < 0.001$
2	$t_{49}^{2} = -9.355$	$p < 0.001$
4	$t_{49}^{4} = -7.091$	$p < 0.001$
6	$t_{49}^{6} = -5.730$	$p < 0.001$
8	$t_{49}^{8} = -1.456$	$p = 0.149$
10	$t_{49}^{10} = -0.334$	$p = 0.739$

REFERENCES

Bays, P. M., Catalao, R. F. G., and Husain, M. (2009). The precision of visual working memory is set by allocation of a shared resource. *Journal of vision* 9 10, 7.1–11

Berens, P. (2009). Circstat: A matlab toolbox for circular statistics. *Journal of Statistical Software* 31, 1–21. doi:10.18637/jss.v031.i10

Georgopoulos, A., Kalaska, J., Caminiti, R., and Massey, J. (1982). On the relations between the direction of two-dimensional arm movements and cell discharge in primate motor cortex. *Journal of Neuroscience* 2, 1527–1537. doi:10.1523/JNEUROSCI.02-11-01527.1982

Schneegans, S. and Bays, P. M. (2016). No fixed item limit in visuospatial working memory. *Cortex* 83, 181–193. doi:https://doi.org/10.1016/j.cortex.2016.07.021
